# Automated Prediction of Preferences Using Facial Expressions

**DOI:** 10.1371/journal.pone.0087434

**Published:** 2014-02-04

**Authors:** David Masip, Michael S. North, Alexander Todorov, Daniel N. Osherson

**Affiliations:** 1 Estudis d'Informatica Multimedia i Telecomunicacions, Universitat Oberta de Catalunya, Barcelona, Spain; 2 Computer Vision Center, Universitat Autonoma de Barcelona, Barcelona, Spain; 3 Department of Psychology, Columbia University, New York, New York, United States of America; 4 Department of Psychology, Princeton University, Princeton, New Jersey, United States of America; Universidad de Castilla-La Mancha, Spain

## Abstract

We introduce a computer vision problem from social cognition, namely, the automated detection of attitudes from a person's spontaneous facial expressions. To illustrate the challenges, we introduce two simple algorithms designed to predict observers’ preferences between images (e.g., of celebrities) based on covert videos of the observers’ faces. The two algorithms are almost as accurate as human judges performing the same task but nonetheless far from perfect. Our approach is to locate facial landmarks, then predict preference on the basis of their temporal dynamics. The database contains 768 videos involving four different kinds of preferences. We make it publically available.

## Introduction

Recently, social psychologists have shown that people can infer which of two stimuli are preferred by human observers just by viewing covertly recorded videos of the observers’ faces [1], [2]. Automating these inferences might be useful to the development of electronic devices that respond in human-like ways to their users. Previous research related to this goal has involved face recognition [3], social trait inference [4]–[7], and the analysis of expression [8], [9], but not the prediction of preference from spontaneous videos. Previous work on the automated analysis of facial expressions, moreover, tends to focus on the six basic emotions defined by [10], and the Facial Action Coding System [11]. These studies are mainly limited to exaggerated expressions with posed dynamics. Likewise, publically available face data typically involve exaggerated facial expressions. We propose here to study more mundane stimuli, using low resolution videos acquired in a spontaneous and non-controlled setting. The resulting facial expressions are briefer and vastly more challenging to interpret. Specifically, the present paper makes three contributions. (i) We introduce the problem of automated inference of preferences from videos, (ii) we make available an annotated data set (with frame-by-frame landmark locations) for experimental purposes, and (iii) we propose two simple algorithms (as a baseline) for predicting preferences. Our goal is merely to articulate and illustrate the problem of interpreting spontaneous faces rather than to explore the space of possible algorithms.

## Methods

### Database Creation

[1] created a video database divided into four categories: *people*, *cartoons*, *animals*, and *paintings*. Eight subjects examined twelve pairs of images from each category. The two images in a pair were examined serially. When viewing people, they judged which of the two was more attractive. When viewing cartoons, they judged which was funnier. When viewing animals, they judged which was cuter, and when viewing paintings they judged which was aesthetically superior. For details about counterbalancing and experimental design, see [1]. Unknown to the subjects, their faces were covertly recorded while they examined a given pair of images. Only after both images in a given pair were shown and withdrawn did the subject indicate his/her preference; hence, recording occurred while the face was involved in nothing more than examining an image. The recording of the videos was approved by the Institutional Review Board (IRB) of Princeton University, and participants signed a film release authorizing the use of the data for future studies.

In a second phase, 56 new participants tried to guess the original subjects’ preferences about the pairs of images just by observing their faces. The second set of subjects did not have access to the pairs of images shown earlier; they made their guesses about preference based only on videos of faces. Henceforth, following the terminology of [1], we call the first set of subjects “targets” and the second second set “perceivers.” Each target was viewed by 14 perceivers, drawn from the set of 56.

The total number of videos in the experiment is 768 (4 categories × 8 targets × 12 pairs of videos × 2). In this paper we consider video pairs as the basic processing unit, yielding 96 pairs for each category. Individual videos lasted three seconds for the people, paintings and animal stimuli, and seven seconds for the cartoons. All videos were recorded at a rate of 24 frames per second; they were acquired via WebCam with 640×480 RGB resolution. The entire data base is available at http://tlab.princeton.edu/databases/ (Princeton Preferences from Facial Expressions Data Set).

### Facial Landmark Detection

**Figure 1:**
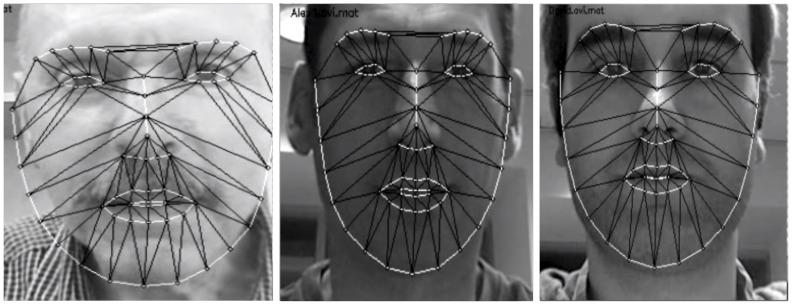
Examples of landmarks assigned to faces. Localization of the landmark points were fitted on the authors pictures (for illustrative purposes).

**Figure 2:**
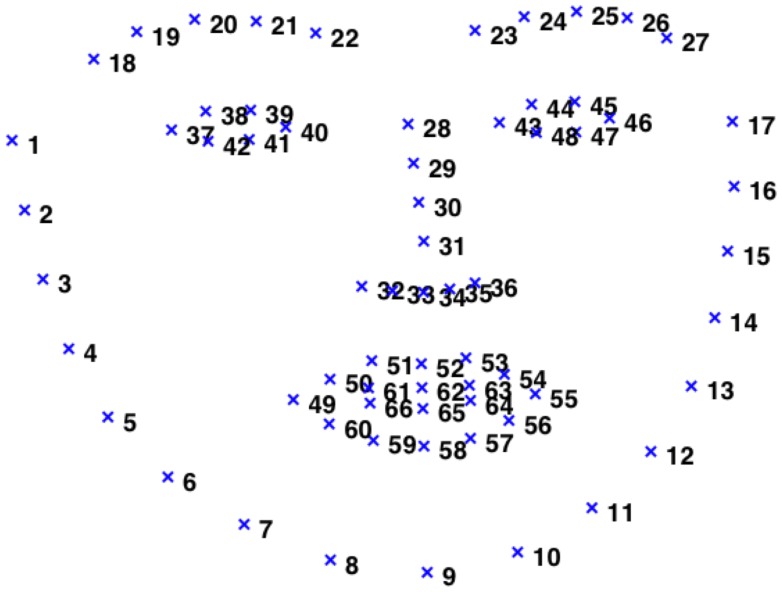
The numbering of the 66 landmarks on a typical face.

Our algorithm relies on the dynamics of salient points that reveal the structure of faces. These points are called “landmarks.” Most algorithms for landmark identification focus on local, nonoverlapping regions of the face [12] or else create a joint distribution of potential landmarks over the whole face [13]. Here we rely on the distribution approach developed by [14]. This algorithm is fast (usable in real time), and its source code is publically available. Given the relatively low quality of our videos, it was necessary to modify the original code to improve the localization of the face in the image. A recently trained version of the [15] face detector algorithm was used for this purpose. Sixty-six landmarks were extracted from each frame. Figure 1 provides examples, and Figure 2 shows the landmark numbering.

**Figure 3:**
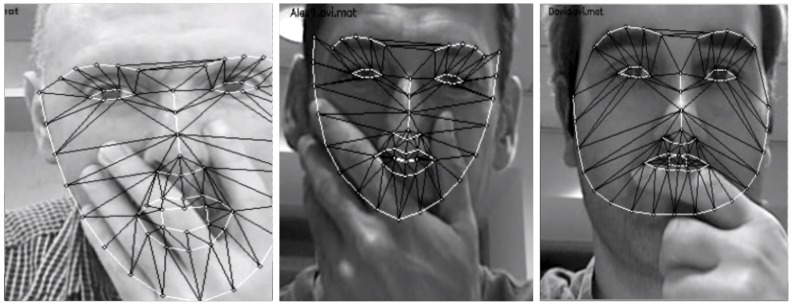
Examples of landmark distortion due to partial occlusion. Given that the participants were unaware of being recorded, some videos presented occlusions that prevented their further processing. The figure shows examples of these distortions on authors’ pictures for illustrative purposes.

As noted above, the eight targets (i.e. the subjects in the first phase of the experiment) were recorded covertly. As a consequence, some of the videos suffered from occlusions (e.g., a hand over the mouth) that made them problematic for the analysis of facial expression; see Figure 3 for examples. Relying on visual inspection, we eliminated all pairs of videos in which one or both included such defective frames; in addition one target was eliminated because she chewed gum throughout the experiment. The last row of Table 1 displays the number of surviving video pairs for each category.

**Table 1:** Percent accuracy on the four domains.

	People	Cartoons	Paintings	Animals
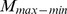	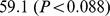	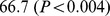	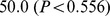	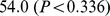
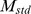	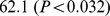	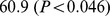	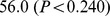	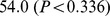
JESP	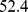	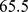	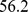	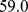
# Videos pairs	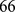	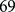	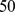	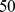

### Normalization Process

After pruning the data (as above) and performing landmark detection, each frame was normalized via the following procedure. First, the coordinates of the center pixel in each eye were computed as the mean of the six corresponding landmarks (37 to 42 for the left eye, and 43 to 48 for the right eye). All landmarks were then rigidly displaced so that the center of the left eye had coordinates (100,100). Second, the inter-eye distance 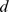 was computed and all landmark coordinates were multiplied by 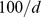. This sets the inter-eye distance to 100 pixels.

The beginning and end of a video often displayed exaggerated mobility and movement. This might be due to the cognitive resources needed to engage the task when the image appears, and to disengage when a judgment is reached. To obtain greater stability, we analyzed just the middle third of each video, discarding frames from the first and last thirds. Other ways of defining a video’s “middle” section (e.g., by discarding frames from just the first and last quarters) yield similar results to those reported below. The use of thirds struck us as the most natural strategy, and we did not attempt to maximize our accuracy by choosing the boundaries accordingly.

Finally, we noticed greater facial mobility to *un*attractive stimuli in the *people* task, and to *non*cute images in the *animals* task. In the experiment [9], preferences were solicited on the basis of attractiveness and cuteness (not their reverse). We therefore switched the sense of preferences in these two domains (both involving the appeal of animate stimuli), and attempted to predict which face in a video pair expressed *less* preference for its stimulus. Specifically, we hypothesized that greater mobility would occur in target faces exposed to the *less* appealing stimulus in a pair. This reversal is left implicit in what follows.

### Video Descriptors and Statistical Algorithms

For the data defined above, the goal of a candidate algorithm is to predict which of the two videos in a given pair is associated with preference (e.g., shows the target when s/he is viewing a cartoon that s/he subsequently designates as funnier than the alternative).

Our strategy is to compute a certain statistic for each video then predict the preference-video to be the one with higher value on the statistic. Two statistics were defined for this purpose; each is a plausible measure of the mobility of the face. To describe the two measures, let a video be composed of 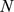 frames, 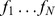. For each frame 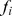, define the *center* of 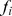 as the average 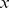
- and 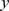-coordinates of the 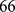 landmarks appearing in 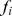. Define the *dispersion* of 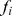 to be the average distance of the 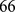 landmarks to the center. We measured variation in dispersion through time via the following statistics.


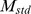
, the standard deviation of the set of dispersions manifested in the frames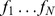.


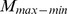
, the difference between the maximum and minimum dispersions manifested in the frames 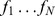.

We hypothesize that the video with more dispersion corresponds to the preferred picture (cartoon, etc.). Note that 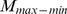 is better able to exploit brief, extreme gestures (involving just a few frames) but is sensitive to noise in the landmark locations. 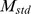 is more noise resistant because every frame contributes to its value. It is easily verified that the two measures are correlated insofar as the dispersion of the landmarks in time has a Gaussian distribution. Notice that the algorithms based on these statistics do not exploit the temporal order of the frames 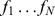.

## Results of Statistical Algorithms

For each of the four domains, Table 1 shows the percent of video pairs that 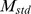 and 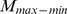 accurately label. We did not apply learning in this first baseline experiment. Instead, each video is predicted as the chosen one if the value of the single statistic (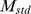 or 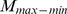 ) is the highest in the pair. The ground truth labels are the original choices of the target participants. To illustrate, 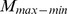 correctly labeled two thirds of the cartoons. As a comparison, we computed the probability of obtaining the same or greater success by throwing a fair coin in response to each pair of videos. For example, the probability of such a coin-flipper reaching at least the level of accuracy shown by 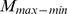 on Cartoons is only 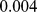 (via a binomial test). Pooling all 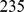 pairs of videos across the four domains, 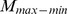 correctly classified 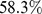 (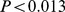) and 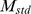 correctly classified 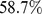 (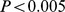).

The row labeled “JESP” in Table 1 shows the results obtained by the human perceivers studied in [1]. The row is relative to just the 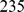 pairs of videos that are free of occlusions and gum-chewing. Performance is similar when all 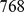 videos are included (as in [1]); with all the data, accuracy is 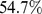, 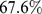, 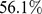 and 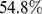 for the four domains, respectively.

Overall, the table reveals better-than-chance performance by 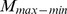 and 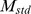 for *people* and *cartoons* but scant accuracy for *paintings* and *animals*. Human perceivers do not perform much better than these simple algorithms. To explore the matter further, for each of the 235 pairs of videos, we define the *human accuracy* for that pair to be the percentage of correct classifications on the part of the fourteen perceivers who evaluated that pair. Likewise, we define the 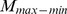 *difference score* to be the difference between the 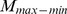 score on the first minus the second videos – and similarly for 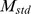. The correlation between human accuracy and the 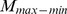 difference score is only 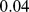; for 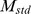 it is only 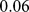. These low correlations suggest little agreement between human and algorithmic inferences. In turn, the low agreement suggests the possibility of designing algorithmic predictors of preference that are more accurate than those offered here.

## SVM Classification and Results

We next sought to determine whether prediction can be improved by submitting the data to a learning algorithm. Instead of using a single value to describe the average dispersion of the landmarks, we compute the proposed descriptors (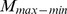 and 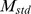 ) on each landmark independently. We allow the learning algorithm to weight the contribution of each landmark to the preference prediction. From this perspective we consider each of the 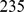 pairs of videos to be a sample in a classification problem. The label on a given sample is either 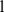 or 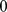 depending on whether the first or second video shows the target’s preference-face. For each pair of videos, we constructed a feature vector for that pair via the following procedure. Let individual video 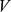 be composed of 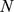 frames, 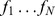.

- Compute the center 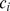 of each frame 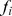 as the average 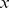
- and 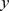-coordinates of the 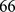 landmarks in 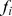.
- For each landmark 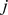 in frame 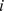, compute the Euclidean distance from 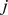 to the frame-center 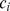. Gathering these computations for landmark 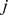 across the frames 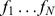 yields a real vector of length 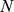; the vector records the changing distances between 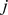 and the frame centers 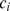. There are 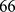 such vectors, one for each landmark.
- For each of the 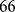 vectors, compute the difference between its maximum and minimum value across the 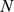 frames. In the same way, for each of the 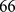 vectors compute the standard deviation of its values. Concatenating the two resulting vectors – 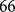 max-min statistics followed by 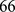 standard deviations – yields a 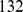-dimensional feature vector 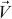 for the starting video 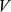.
- Given a pair 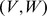 of videos, the feature vector for the pair is defined to be 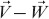, the coordinate-wise difference between the features of 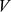 and 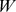.

Relying on these features, a nonlinear Support Vector Machine (SVM) with a Radial Basis Function (RBF) kernel [16], [17] was applied as a classification rule on the video pairs available in each of the four domains separately. We executed 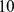 random iterations of a 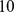-fold cross validation protocol to assess the results. Folds were constructed balancing the number of samples from each class. The dimensionality of the data was reduced by applying Principal Component Analysis on the training set (preserving 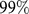 of the variance). In order to estimate the parameter 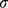 (for the RBF Kernel) and the soft margin 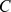 (for SVM), only the training data were used. The 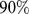 of the data reserved for training was split into two subsets, 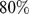 for internal training and 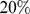 for internal validation. The SVM/RBF algorithm was then applied to the 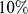 testing data, using the two fixed parameters. Table 2 shows the results of 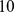 applications of the algorithm in this way. It can be seen that predictive accuracy is only slightly higher than for 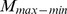 and 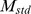 (applied without training).

**Table 2:** Results using SVM/RBF: mean accuracies and 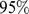 confidence intervals.

	People	Cartoons	Paintings	Animals
SVM-RBF	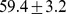	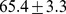	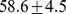	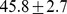

## Conclusion

In this paper we introduce the problem of automatically inferring preferences from spontaneous facial expressions. We make available an annotated database, and propose baseline methods to infer preferences. The simple descriptors 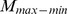 and 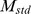 perform better than chance in two domains (*people*, *cartoons*), and at approximately the same modest level as human perceivers. Classification based on a standard learning algorithm yields only limited improvement. The question immediately arises whether the faces in [1] hold further information that can be exploited to reveal preference. Developing more successful algorithms than ours would provide an affirmative answer. Failure would suggest that faces are often opaque, and it would invite hypotheses about which social circumstances allow more emotional information to invade the face. Research in this area provides a rare point of convergence between Computer Science and Social Psychology.
